# Supplementary material for: On-the-fly closed-loop materials discovery via Bayesian active learning
Source: Nat Commun. 2020 Nov 24;11:5966. doi: 10.1038/s41467-020-19597-w (PMC7686338; doi:10.1038/s41467-020-19597-w)
Supplement: Supplementary file 1 — Supplementary Information [file 41467_2020_19597_MOESM1_ESM.pdf]

Supplemental Information

Title: On-the-fly Closed-loop Autonomous Materials Discovery via Bayesian Active Learning

Authors: A. Gilad Kusne[‡,1,2,*], Heshan Yu[‡,2], Changming Wu[3], Huairuo Zhang[4,5], Jason Hattrick-Simpers[1], Brian DeCost[1], Suchismita Sarker[6], Corey Oses[7], Cormac Toher[7], Stefano Curtarolo[7], Albert V. Davydov[4], Ritesh Agarwal[8], Leonid A. Bendersky[4,5], Mo Li[3], Apurva Mehta[6], Ichiro Takeuchi[2,9,*]

Affiliations:

[1] Materials Measurement Science Division, National Institute of Standards and Technology, Gaithersburg, MD 20899, US

[2] Materials Science and Engineering Department, University of Maryland, College Park, MD 20742, US

[3] Electrical & Computer Engineering Department, University of Washington, Seattle, WA 98195, US

[4] Materials Science and Engineering Division, National Institute of Standards and Technology, Gaithersburg, MD 20899, US

[5] Theiss Research, Inc., La Jolla, CA 92037, US

[6] Stanford Synchrotron Radiation Lightsource, SLAC National Accelerator Laboratory, Menlo Park, CA 94025, US

[7] Mechanical Engineering and Materials Science Department and Center for Autonomous Materials Design, Duke University, Durham, NC 27708, US

[8] Materials Science and Engineering Department, University of Pennsylvania, Philadelphia, PA 19104, US

[9] Maryland Quantum Materials Center, University of Maryland, College Park, MD 20742, US

* Emails: aaron.kusne@nist.gov; takeuchi@umd.edu

‡ These authors contributed equally to this work.

This document contains the Tables and Figures associated with the Methods section.

Supplementary Table 1. Knowledge and Control implemented in CAMEO.

Knowledge and Control	CAMEO
Knowledge: Past experiments both physical and computational	Automated access to experimental and density functional theory materials structure databases. Includes Inorganic Crystal Structure Database and AFLOW.org.
Knowledge: Materials physics theory	Phase mapping and structure theory including Gibbs phase rule via constraint programming
Knowledge: Materials synthesis and processing	NA
Knowledge: Measurement science	X-ray diffraction simulation capability using structure data as input
Control: Synthesis control	NA
Control: Characterization	X-ray diffraction: high-throughput X-ray diffraction system[1] at the Stanford Synchrotron Radiation Lightsource (SSRL) and Bruker D-8*
Control: Communication	GUI for user interface; Interface to databases to store and share knowledge with experts and other AIs; Network interface for instrument control

Supplementary Table 2. List of physical constraints in [(32)] method and associated encoding methods.

Physical Constraint	Encoding Method
Phase regions are cohesive and phase boundaries are continuous	1. If two or more set of vertices share the same phase region label but are not connected by vertex neighbors, differing labels are assigned to the disconnected sets.<br>2. The Markov Random Field smoothness constraint
Materials of similar synthesis and processing parameters have similar properties	1. Markov Random Field smoothness constraint<br>2. Harmonic Energy Minimization for label propagation
Abundances of phases is non-negative	Karush–Kuhn–Tucker conditions[2]
X-ray diffraction intensity is non-negative	Karush–Kuhn–Tucker conditions[2]
Soft Gibbs Phase Rule - Upper bound limit on number of constituent phases	Upper limit on number of endmember limits allowed in each phase region
Identified endmembers should be physically realizable	Volume constraint on identified / predicted endmembers

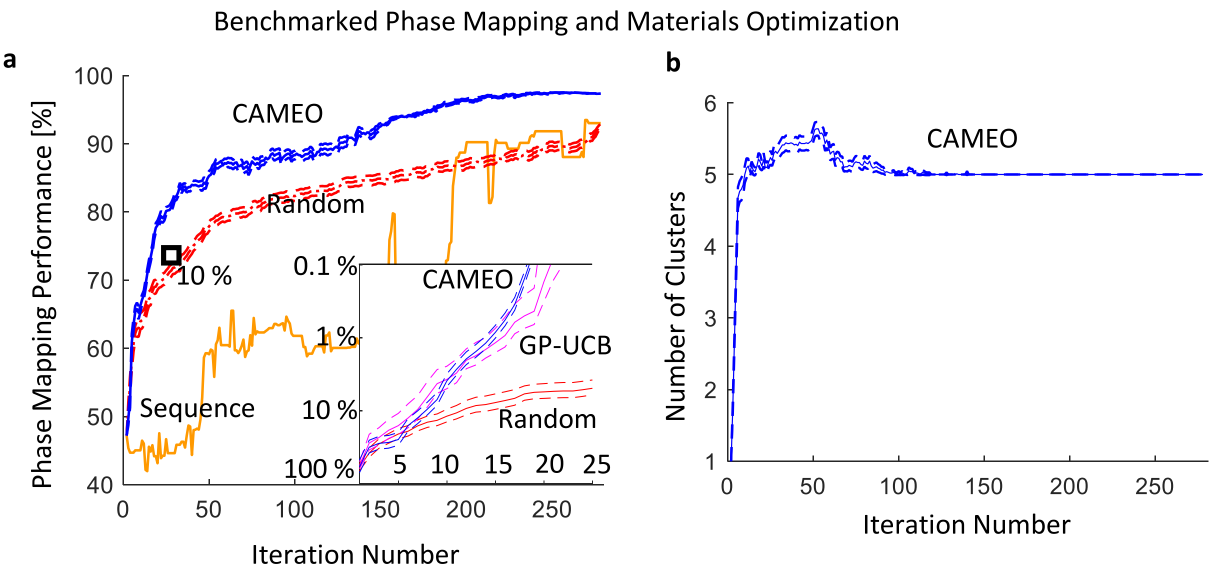

Supplementary Figure 1. Benchmarking performance. a) Main figure: Phase mapping performance demonstrating that CAMEO provides a significant advantage over the three alternatives: random sampling, sequential sampling, and measuring 10 % of the samples well distributed over the composition space. Subset figure: Material optimization performance. The benchmark materials optimization challenge is highly simple with a very prominent, broad peak – a challenge that Bayesian optimization schemes excel at. Nevertheless, CAMEO provides improved results over the next best alternative, GP-UCB. Of note is CAMEO's initial lag in performance due to its initial goal of maximizing phase mapping performance. Once phase mapping performance converges, it then switches to materials optimization and shows faster performance than GP-UCB. b) The number of clusters for the benchmark dataset was initialized to 5 and while this number on average increased during CAMEO's phase mapping, it converged to 5. Demonstrating that improved performance was not due to increased complexity defined by a larger number of clusters.

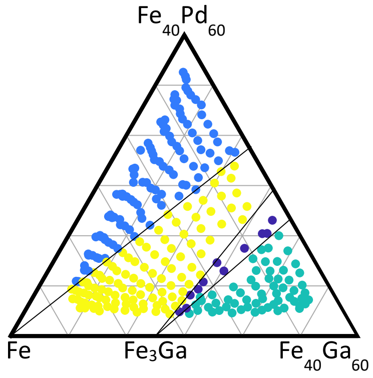

Supplementary Figure 2. Color coded phase map prior derived from AFLOW.org computed tie-lines (black lines) for the benchmark Fe-Ga-Pd material system.

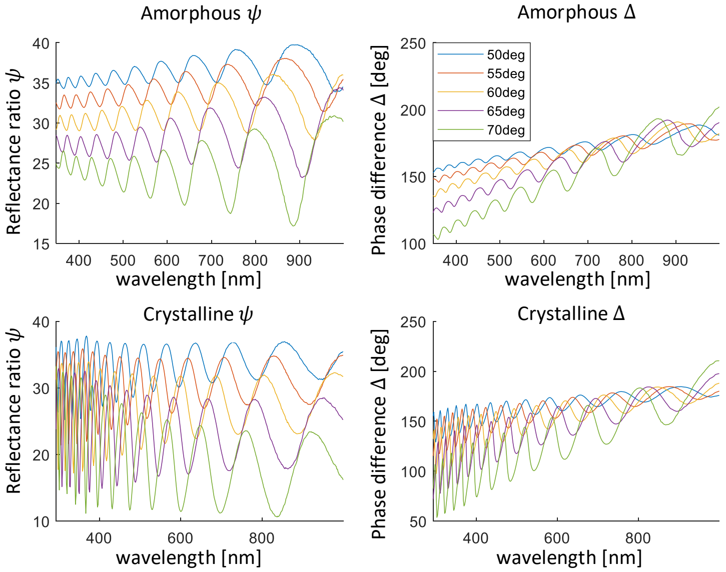

Supplementary Figure 3. Example Ge-Sb-Te optical data used for phase mapping prior.

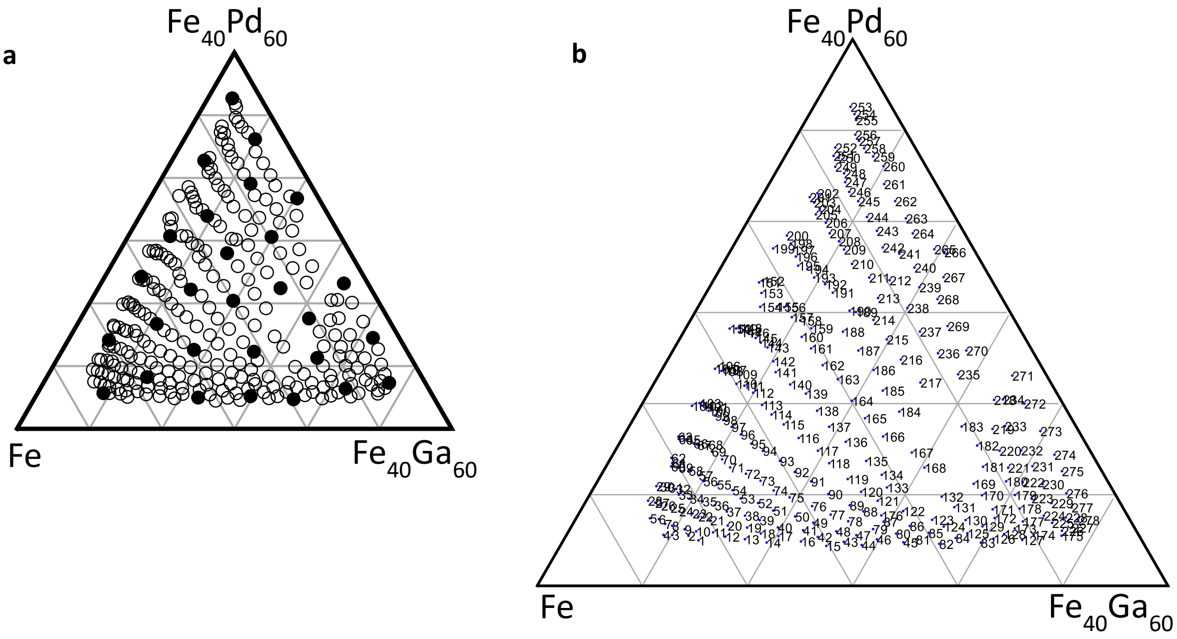

Supplementary Figure 4. a) For the 10 % material selection out of the 278 materials in the composition spread, the selected 28 materials are indicated with black filled circles. b) The order of materials measured during sequential measurement.

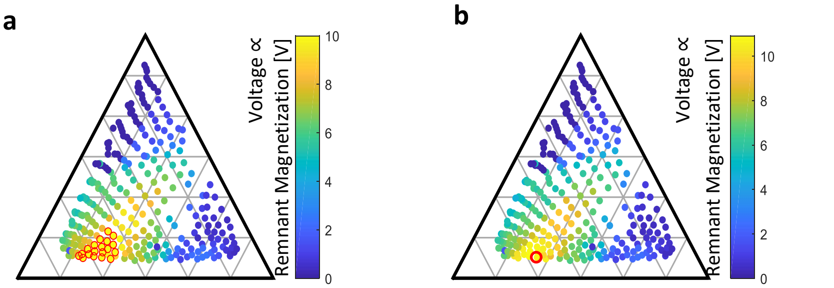

Supplementary Figure 5. Modifications made to the Fe-Ga-Pd remnant magnetization as measured as scanning SQUID voltage signal. a) Red circles indicate the samples with saturated voltage of 10 V, b) Modified voltage by enhancing main voltage peak at $\mu \quad = Fe_{78}Ga_{16}Pd_6$ and the maximum indicated with a red circle.

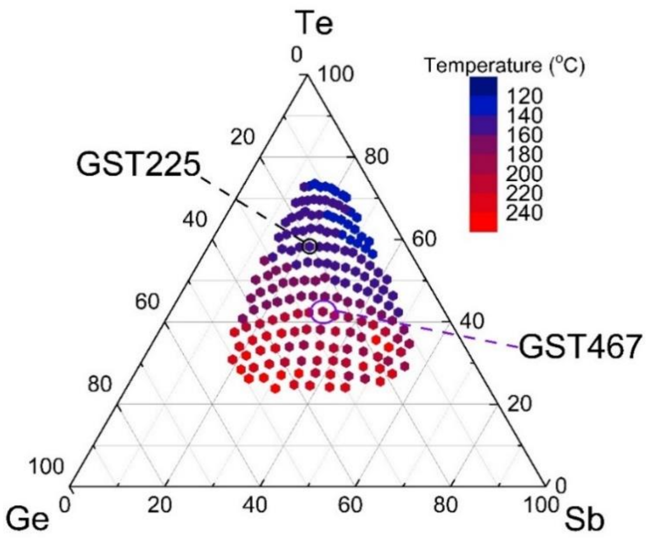

Supplementary Figure 6. Phase-change temperature mapping of the combinatorial Ge-Sb-Te spread.

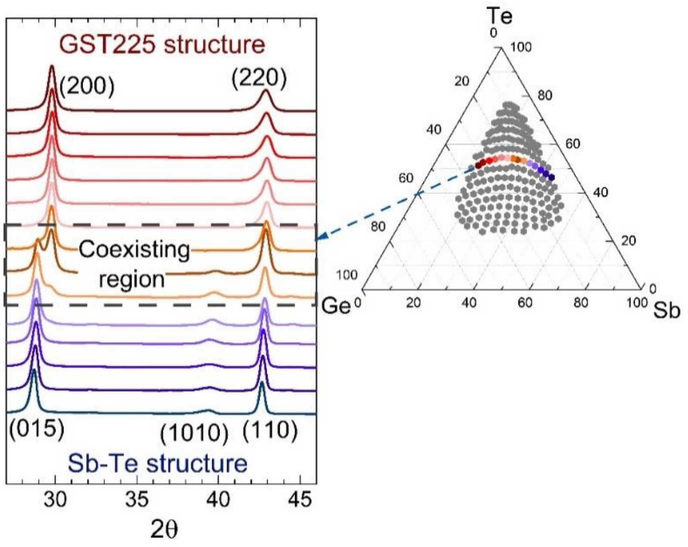

Supplementary Figure 7. Structural evolution FCC-Ge-Sb-Te (GST) structure (top) to the Sb-Te structure (bottom) across the line of composition marked in the phase diagram on the right. Peak indices are denoted.

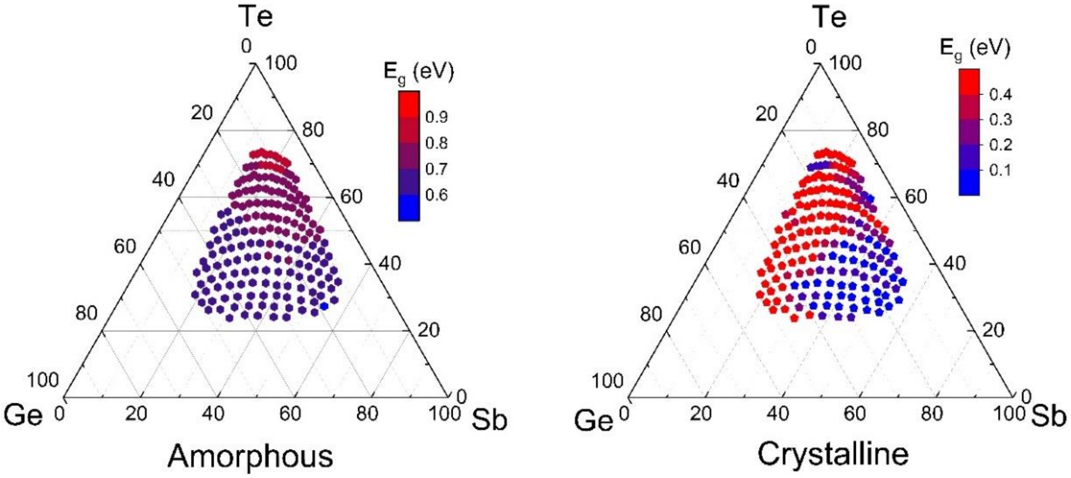

Supplementary Figure 8. The optical bandgap of amorphous (left) and crystalline (right) states for a combinatorial Ge-Sb-Te spread.

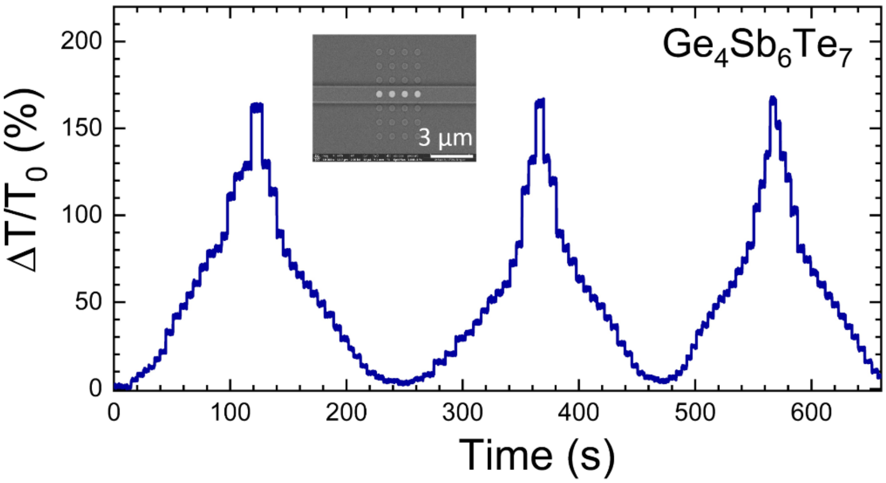

Supplementary Figure 9. The performance of the photonic device fabricated by the new nanocomposite PCM, GST467. The symmetric multi-level switching is realized. The inset is the top view of the photonic device used for multi-level switching, endurance test and comparison between $Ge_4Sb_6Te_7$ and $Ge_2Sb_2Te_5$.

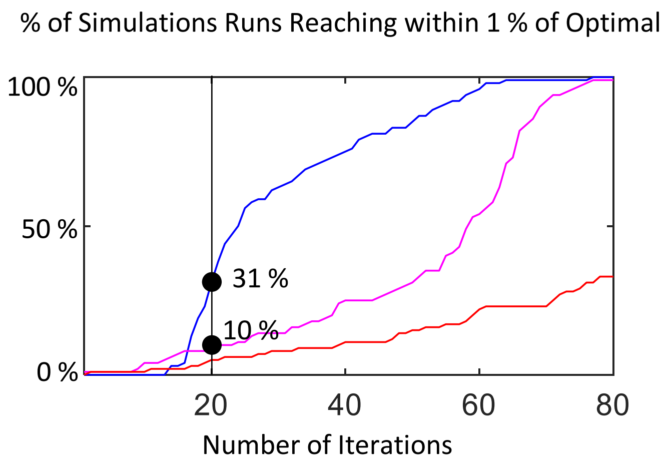

Supplementary Figure 10. Comparison of CAMEO (blue), GP-UCB (magenta), and random (red) for materials optimization. Each curve shows the percentage of the 100 (post data collection) simulation runs for each active learning scheme which have reached within 1 % of the optimal ΔE_g. Within the first 20 iterations, 31 % of CAMEO's runs have reached within 1 % of the optimal ΔE_g, compared to 10 % for GP-UBC.

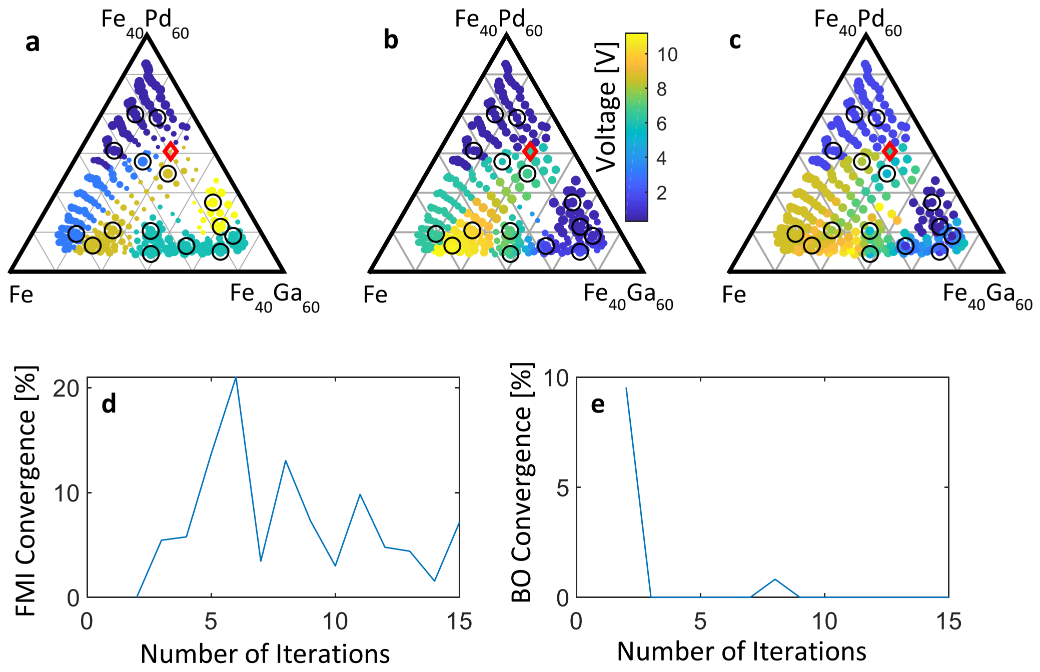

Supplementary Figure 11. Live visualizations for the Fe-Ga-Pd system. a) Samples labeled with the same color are identified to belong to the same phase region. The size of the filled circles indicates probability of estimated region label. Regions of small circles indicate high likelihood of phase region boundary. Black circles indicate samples that have been measured for x-ray diffraction and target functional property. Red diamond indicates CAMEO's selection of the next sample to measure. b) Color indicates CAMEO prediction of functional property, with each phase region fitted with its own Gaussian process. Here the measured voltage is the signal from scanning SQUID and is proportional to magnetization. c) Color indicates computed GP-UCB acquisition function for (c) d) FMI phase mapping convergence calculated between each subsequent iteration. e) Convergence in maximum functional property value identified computed as the difference in the max identified property in consecutive iterations.

Supplemental References

1. Gregoire, J. M. et al. High-throughput synchrotron X-ray diffraction for combinatorial phase mapping. J. Synchrotron Radiat. 21, 1262–1268 (2014).

2. Kuhn, H. W., et al., Nonlinear Programming, Traces and emergence of nonlinear programming, pp. 247–258 (Springer, 2014).
